# Thermomechanical and Morphological Properties of Poly(ethylene terephthalate)/Anhydrous Calcium Terephthalate Nanocomposites

**DOI:** 10.3390/polym12020276

**Published:** 2020-01-30

**Authors:** Franco Dominici, Fabrizio Sarasini, Francesca Luzi, Luigi Torre, Debora Puglia

**Affiliations:** 1 Civil and Environmental Engineering Department, University of Perugia, Strada di Pentima 4, 05100 Terni, Italy; franco.dominici@unipg.it (F.D.); francesca.luzi@unipg.it (F.L.); luigi.torre@unipg.it (L.T.); 2 Department of Chemical Engineering Materials Environment, Sapienza-Università di Roma and UdR INSTM, Via Eudossiana 18, 00184 Roma, Italy; fabrizio.sarasini@uniroma1.it

**Keywords:** recycled poly(ethylene terephthalate), rPET, Calcium terephthalate salts, high performance nanocomposites

## Abstract

Calcium terephthalate anhydrous salts (CATAS), synthetized by reaction of terephthalic acid with metal (Ca) oxide were incorporated at different weight contents (0–30 wt. %) in recycled Poly(ethylene terephthalate) (rPET) by melt processing. Their structure, morphology, thermal and mechanical properties (tensile and flexural behavior) were investigated. Results of tensile strength of the different formulations showed that when the CATAS content increased from 0.1 to 0.4 wt. %, tangible changes were observed (variation of tensile strength from 65.5 to 69.4 MPa, increasing value for E from 2887 up to 3131 MPa, respectively for neat rPET and rPET_0.4CATAS). A threshold weight amount (0.4 wt. %) of CATAS was also found, by formation at low loading, of a rigid amorphous fraction at the rPET/CATAS interface, due to the aromatic interactions (π−π conjugation) between the matrix and the filler. Above the threshold, a restriction of rPET/CATAS molecular chains mobility was detected, due to the formation of hybrid mechanical percolation networks. Additionally, enhanced thermal stability of CATAS filled rPET was registered at high content (T_max_ shift from 426 to 441 °C, respectively, for rPET and rPET_30CATAS), essentially due to chemical compatibility between terephthalate salts and polymer molecules, rich in stable aromatic rings. The singularity of a cold crystallization event, identified at the same loading level, confirmed the presence of an equilibrium state between nucleation and blocking effect of amorphous phase, basically related to the characteristic common terephthalate structure of synthetized Ca–Metal Organic Framework and the rPET matrix.

## 1. Introduction

Disposal of waste materials has become an urgent problem that can be solved by following two general paths: re-using the materials disposed, for suitable applications, as received, or recycling the waste to produce regenerated materials that could be applied again in the same or in another industrial field [1]. For instance, waste poly(ethylene terephthalate) (PET) plastic waste is neither biodegradable nor compostable, and due to its wide use worldwide substantial disposal problems are created. Recycling has emerged as the most practical method to deal with this issue, especially with products such as recycled PET (rPET) beverage bottles or fibers for textiles [2].

On the other hand, although PET is a recyclable polyester, its post-consumer reuse is limited by the weakening of its physical-mechanical properties, due to the reduction of molecular weight and viscosity, derived from the cleavage of the chains during processing [3]. As a solution to this problem, extruded blends with satisfactory properties based on virgin PET, with marginal proportions of rPET, were developed and used at an industrial level. However, it is necessary to increase the use of recycled PET in these mixed formulations to reduce costs and contribute to compliance with increasingly stringent regulations on plastic waste disposal.

To achieve the result of using increasing fractions of rPET without adding virgin material, it is mandatory to increase the mechanical properties of the polymer with the incorporation of low cost, commercially available nanoparticles/nanofillers [4]. There are many examples available in the literature dealing with the selection of unmodified or chemically modified layered nanoclays [5,6,7].

However, even if it was shown that substantial increase of mechanical properties can be achieved by high MMT (montmorillonite) content and optimum strength and stiffness at low level [8], the main limit of the use of organo-modified nanoclays relies in the decomposition of alkylammonium ether that can influence the PET degradation during the processing. A recent paper investigated the use of graphene nanoplatelets [9], demonstrating how the higher-order structure of PET can be tuned by considering different graphene loading. The authors interestingly found that the presence of a significant rigid amorphous fraction (RAF) in the nanocomposites increased with an increase in the graphene loading, while at low loadings the stiffening effect was observed to be quite small in the glassy state region. At a value of 2 wt. %, graphene formed a mechanical percolation network with the RAF of PET and the PET chains were geometrically restricted by the incorporation of graphene, resulting in an unexpectedly higher modulus of nanocomposites both below and above T_g_.

The effect of the nanometric calcium terephthalate anhydrous salts (CATAS) was studied in analogy to the above mentioned PET/GNP (polyethylene terephthalate/graphene nanoparticles) nanocomposites work. The idea is to design a circular use of PET, using terephthalic acid (TPA) recovered from the hydrolysis of PET for the preparation of terephthalate salts of various metals such as aluminum, magnesium, sodium, calcium potassium, etc. [10,11]. The production of these salts with nano-sized lamellar structure can be obtained with appropriate control of the reaction parameters [12,13]. Many different terephthalate salts, or metal–organic framework (MOFs) may be obtained by using sodium, potassium, aluminum, magnesium, calcium and other metals salts [14,15]. They are hybrid materials, composed of metal nodes and coordinating organic linkers, arranged in highly regular motifs that lead to materials exhibiting ultra-high surface areas. Applications are therefore proposed that use this porosity for reversible host–guest behavior, for example, in gas storage, catalysis and drug delivery. Attempts were made to develop existing MOFs and explore new applications using known functionalities, and introducing flexibility, defects and stimuli responsive behavior [16]. The formation of nanocomposites by combining MOFs with more processable materials such as polymers, not only engages with the theme of new materials discovery, but also offers new solutions for the manufacturing of robust bulk structures.

A few examples are available in the scientific literature on the use of these salts in thermoplastic nanocomposites, as in the case of applications that consider terephthalate salts in batteries [17,18,19] or gas separation applications, by taking into advantage of their defined porosity, high surface area, and catalytic activity. A recent review covers various topics of MOF/polymer research, where MOF/polymer hybrid and composite materials are highlighted, encompassing a range of material classes, such as bulk materials, membranes, and dispersed materials [20].

On the other hand, the chemical compatibility between the terephthalate salts and the polymer molecules, rich in stable aromatic rings, suggested the possibility of investigating the role of these salts in the thermomechanical behavior of recycled PET.

The objective of the present investigation was to study the structure of rPET_CATAS nanocomposites and the effect of nanofillers introduction on mechanical properties. The study was conducted by selecting two levels of loadings (0.1–1 wt. % and 2–30 wt. %), aiming to correlate the internal structure with the bulk thermomechanical performance of recycled PET. We specifically aimed to study these properties from the different perspective of evaluating not only the dispersibility at the macrolevel, but also checking the higher-order structure of the polymeric matrix in presence of the selected nanofillers.

## 2. Materials and Methods

Polyethylene terephthalate (I. V., 0.65 dl/g, moisture content 1.3%, bulk density 250–350 kg m^−3^) was supplied by Extremadura Torrepet (Torremejia, Spain). The recycling process adopted by the company intended to recycle post-consumer food grade poly(ethylene terephthalate) containers to produce recycled PET pellets. A four steps process consisted of in house treatment of PET bottles into hot caustic, drying and extrusion of flakes into pellets under vacuum, continuous feeding to a reactor for crystallization at high temperature, under atmospheric pressure for a predefined residence time, after that decontamination by solid state polymerisation process (SSP) is considered. A nanometric metal organic framework consisting of Calcium ions as metal clusters coordinated to terephthalic acid as organic ligand was synthesized in our laboratories to be used as filler in the nanocomposites. Calcium oxide, terephthalic acid, ammonia and water chemical reagents were supplied by Sigma Aldrich (Milan, Italy). The synthesis method is briefly described in the next section.

### 2.1. Preparation of Terephthalate Salts

Powdered terephthalic acid (TPA) was solubilized in an alkaline solution of water and ammonia with stirring at 80 °C for 1 h. Then, calcium oxide was added in a stoichiometric proportion to the acid according to Reaction (1):

C_8_H_6_O_4_ + CaO + 2 H_2_O → C_8_H_4_O_4_Ca • 3 H_2_O (1)

The solution was kept under stirring at 80 °C for 30 min, after that insoluble calcium terephthalate precipitated forming a whitish deposit on the bottom. The solution was filtered, and the solid residue subjected to appropriate washings to eliminate unwanted reaction residues. At the end of the synthesis, calcium terephthalate trihydrate is obtained. To eliminate both moisture and water-bound molecules, the product was kept in a vacuum oven for 2 h at 190 °C. Finally, anhydrous calcium terephthalate (CATAS) was pulverized and sieved to eliminate any conglomerates of salts. 

### 2.2. Production of Composite Materials

Composite materials were manufactured using the melt mixing method by blending the recycled polyethylene terephthalate matrix with the calcium terephthalate salts as shown in Table 1. A co-rotating twin-screw extruder, Microcompounder 5 & 15cc by DSM (DSM explorer 5&15 CC Micro Compounder, Xplore Instruments BV, Sittard, The Netherlands) was used for the mixing at 90 rpm for 60 s by setting a temperature profile of 250–255–265 °C in the three heating zones from top to die. A Micro Injection Molding Machine 10cc, Xplore line by DSM (DSM explorer 5&15 CC Micro Compounder, Xplore Instruments BV, Sittard, The Netherlands) was used to produce the samples according to the standards for flexural and tensile tests. An adequate pressure/time profile was used for the injection of each type of samples, while barrel and mold temperatures were set at 280 and 120 °C, respectively.

### 2.3. Characterization of Terephthalate Salts and rPET/CATAS Nanocomposites 

Morphological characterization of salts and rPET/CATAS nanocomposites was carried out using a field emission scanning electron microscope (FESEM) Supra 25 by Zeiss (Oberkochen, Germany) taking micrographs with an accelerating voltage of 5 kV at different magnifications. Previously, the samples were gold sputtered to provide electric conductivity.

X-ray diffraction (XRD) analysis was performed with a diffractometer X’Pert PRO by Philips (Malvern Panalytical Ltd., Malvern, UK) (CuKα radiation = 1.54060 Å) at room temperature. XRD patterns were collected in the range of 2θ = 10–80° with a step size of 0.02° scan and a time per step of 34 s.

Thermal characteristics of rPET/CATAS nanocomposites were investigated with a temperature-modulated differential scanning calorimeter (MDSC) Q200 by TA Instruments (TA Instrument, Q200, New Castle, DE, USA). A heating scan between 0 and 300 °C was performed at a rate of 10 °C/min in nitrogen flow at 60 mL/min. A three phases model was adopted to describe the higher-order structure of rPET/CATAS nanocomposites. It was demonstrated that sometimes ∆C_p_ at T_g_ in semicrystalline polymers results inconsistent with the amorphous fraction (X_a_), if calculated as complement to one of the crystalline fraction (X_c_). In this case, it is possible to calculate the amorphous fraction with the variation of thermal capacity at T_g_ and a total rigid fraction (X_f_ = 1 − X_a_) that consists of the crystalline phase and another fraction of a phase which remains rigid beyond the glass transition region. Since this rigid phase cannot be detected by ∆C_p_ at T_g_ that is not properly amorphous phase and it does not have a crystalline structure, it was defined rigid amorphous phase X_raf_:

(2) $$X_{a}=\frac{∆C_{P-\mathrm{meas}}^{∆\mathrm{Tg}}-{\;w}_{\mathrm{CATAS}\;}*∆C_{P-\mathrm{CATAS}\;}^{∆\mathrm{Tg}}}{∆C_{P-\mathrm{aPET}}^{∆\mathrm{Tg}}}\;=\;\frac{∆C_{P-\mathrm{PET}}^{∆\mathrm{Tg}}}{∆C_{P-\mathrm{aPET}}^{∆\mathrm{Tg}}}\;=\;1\;-\;X_{f}$$

(3) $$X_{a}=\;1\;-\;(X_{c}+X_{\mathrm{raf}})$$

where $∆C_{P-\mathrm{meas}}^{∆\mathrm{Tg}},∆C_{P-\mathrm{CATAS}\;}^{∆\mathrm{Tg}},∆C_{P-\mathrm{aPET}}^{∆\mathrm{Tg}}$ are ΔC_p_ at T_g_, respectively, for measured nanocomposites, CATAS and 100% amorphous neat PET, while w_CATAS_ is the weight fraction of the salts in the nanocomposites. It is assumed that $∆C_{P-\mathrm{aPET}}^{∆\mathrm{Tg}}=\;$ 0.405 J g^−1^ K^−1^ and heat of melting of 100% crystalline PET is $∆H_{m-\mathrm{cPET}}^{0}=$ 140 J g^−1^. [21].

Thermogravimetric analysis (TGA) on nanocomposites samples was carried out using Seiko Exstar 6300 from Seiko instruments (Seiko, Tokyo, Japan). Thermograms obtained from TGA analysis provides the information related to the thermal stability of material. About 10–15 mg of the sample was subjected for TGA analysis, performed on the samples between room temperature and 900 °C with heating rate of 10 °C/min in nitrogen atmosphere.

A universal electronic dynamometer LR30K by LLOYD Instruments (Lloyd Instrument, Segens worth West, Foreham, UK) was used to carry out tensile tests by setting a cross-head speed of 5 mm min^−1^, in accordance with ASTM D638 standard. Three points bending tests were carried out by setting a span between supports of 64 mm and loading at a deflection rate of 2 mm min^−1^, according to the standard ASTM D790. The analysis of mechanical tests data was carried out by using the Nexigen software (Elis (Electronic Instruments & Systems) s.r.l., Rome, Italy).

Dynamic mechanical analysis of the rPET-based nanocomposites was performed with an Ares N2 rheometer (Rheometric Scientific, Reichelsheim, Germany), by testing samples of about 4 × 10 × 40 mm, gripped with a gap of about 20 mm and tested with rectangular torsion at a frequency of 2π rad/s with a strain of 0.05%, with a temperature ramp of 3 °C/min applied in the range from 30 to 180 °C. Storage (G’) and loss moduli (G”) as a function of the temperature were determined.

## 3. Results and Discussion

### 3.1. Characterization of Calcium Terephthalate Salts 

**Figure 1:**
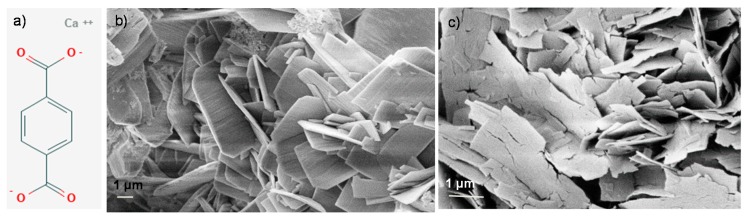
Chemical structure of calcium terephthalate salts (**a**) and FESEM morphology in their hydrated (**b**) and anhydrous state (**c**).

The structural characteristics of terephthalate calcium salts are shown in Figure 1a [22,23,24]. The reaction between terephthalic acid and calcium oxide in water was carried out to produce calcium terephthalate trihydrate salts (CATS) [25]. The production process required an appropriate monitoring of reaction conditions, by acting on the kinetics and using appropriate additives to obtain the nanometric structures. Some attempts were made to optimize the characteristics of the fillers. Lastly, insoluble calcium terephthalate trihydrate salts were obtained by their precipitation in water. A heat treatment at 200 °C for 1 h in a vacuum oven transforms the trihydrate salts (Figure 1b) into the corresponding anhydrous calcium terephthalate (CATAS) salts (Figure 1c): this step was necessary, being the presence of bound water harmful for the melt blending process of the nanocomposites, due to hydrolysis phenomena and formation of cavities within the samples.

Figure 1c shows that after the dehydration (T = 200 °C), the anhydrous salts have cracks with rough surface, revealing that volume contraction happens in the dehydration process [26]. 

**Figure 2:**
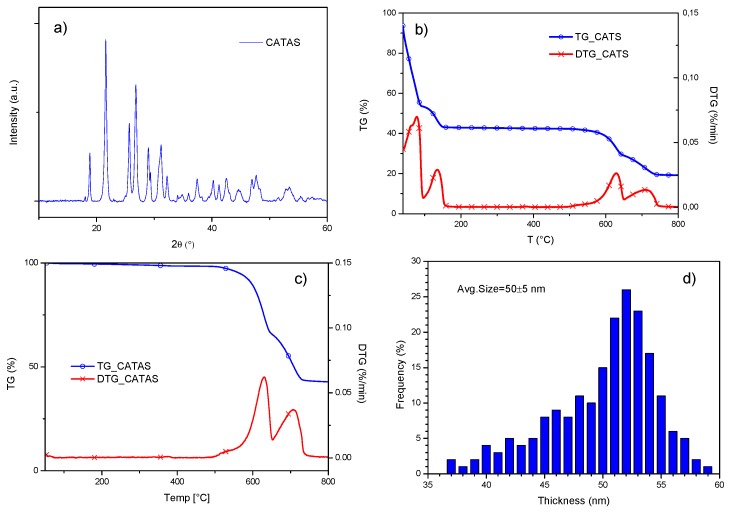
XRD profile for CATAS (**a**); TG/DTG curves for CATS (**b**); and CATAS nanofillers (**c**); thickness distribution for CATAS (**d**).

The X-ray diffraction pattern of CATAS shows diffraction peaks at 18.8°, 21.6°, 25.7°, 26.9°, and 31.2°. According to the report by Mou et al., this structure can be indexed with a space group of C2/c (n. 15) as monoclinic crystal system [17] (Figure 2a). More precisely, the process involves rotation of the ligand, disconnection, and protonation of the carboxylate group on one side and partial disconnection and reformation of Ca–O bond on the other side of the ligand. Mazaj proposes a reversible hydration/dehydration mechanism, which through the breaking of the bonds between Ca^2+^ and carboxylate groups, rotating of ligand and re-coordination of COO^−^ groups to Ca^2+^ centers. The crystal-to-crystal transformations are driven by the tendencies to change the bonding modes between COO^−^ and Ca^2+^ with the change of Ca^2+^ coordination number. Thus, the decrease of Ca^2+^ coordination number, which is usually a consequence of activation, leads to a structural rearrangement, with expansion or contraction of the pores [27]. 

TG and DTG plots for CATS and anhydrous CATAS (Figure 2b,c) showed multiple decomposition steps. In the case of CATS, the first peak at a temperature lower than 100 °C refers to the loss of hygroscopic water and any residual volatile additives present during the reaction. The second peak, at a temperature between 100 °C and 170 °C, refers to the loss of molecular water since a weight loss of 22% (moisture free weight) corresponds to three water molecules per formula unit. The decomposition of the acid salts involved the breaking of the carboxyl groups, the formation of carbonates, and the release of gaseous products. The subsequent mass losses are due to thermal destruction of organic ligands over 500 °C. Figure 2c shows that in the case of anhydrous salts, the main weight loss can be detected between 500 and 800 °C that corresponds to the decomposition of the organic ligands [25,28]. Evaluation of thickness distribution showed that the average size for anhydrous salts was set at 50 ± 5 nm.

### 3.2. Characteristics of rPET/CATAS Nanocomposites 

#### 3.2.1. DMTA (Dynamic Mechanical Thermal Analysis)

**Figure 3:**
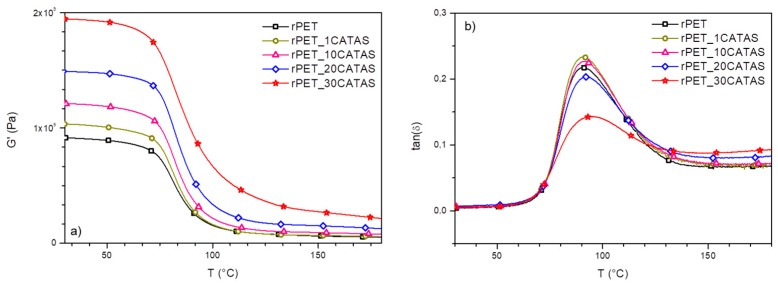
G’ (**a**) and tan δ curve (**b**) from DMTA tests for rPET and rPET/CATAS nanocomposites.

To evaluate the influence of CATAS on viscoelastic behavior of rPET matrix, dynamic mechanical thermal analysis of high loaded rPET nanocomposites was performed. The variation of the storage moduli (G’) and tan δ (G”/G’) with temperature are presented in Figure 3a,b, respectively. The storage modulus behavior in Figure 3a shows that the rPET/CATAS nanocomposites possessed higher storage modulus compared to the reference rPET below and above the T_g_. A gradual increase in storage modulus is observed with increase in CATAS content in rPET matrix (Table 2). Among the nanocomposite samples, rPET_30CATAS had the highest storage modulus compared to reference rPET and other nanocomposite samples. The adsorption of the CATAS onto the macromolecular chains of rPET leads to a constraint in the chains movement and therefore, an improved storage modulus [29], while a sharp decrease in the moduli was observed at the glass–rubber transition at around 90 °C [4]. Regarding the glass temperatures (T_g_), results of tan δ from the DMTA tests can be seen in Figure 3b and Table 2. Compared to rPET, the nanocomposites had the maximum of the peak at slightly higher temperature with increasing content of CATAS temperatures. Thus, the increased value of T_g_ resulting on the addition of nanometric salts confirms that the mobility of polymeric chains was slightly reduced, which in turn affected the flexibility of the samples. On the other hand, the peak values of tan δ increased with an increase in CATAS up to 10% wt., where the influence of the elastic component starts to prevail on the viscous one: at higher filler contents (20–30 wt. %), a decrease in the tan δ is observed, mainly due to a combination of viscoelastic and physical behavior. In details, a prevalent rigid amorphous fraction (X_raf_), formed in the interfacial region between rPET and CATAS, justifies the increase in elastic modulus, so that the nanofiller is able to restrict the molecular movement of the amorphous rPET chains both in the interfacial region and in the amorphous region. This restriction is also evident in the registered G’ values when measured at 150 °C (after Tg) (Table 2): the limited decrease in G’ after the Tg can be mainly related to the CATAS physical interactions that when present in large amount physically constrains the mobility of the polymeric chains.

#### 3.2.2. Mechanical Characterization (Flexural and Tensile Tests)

**Figure 4:**
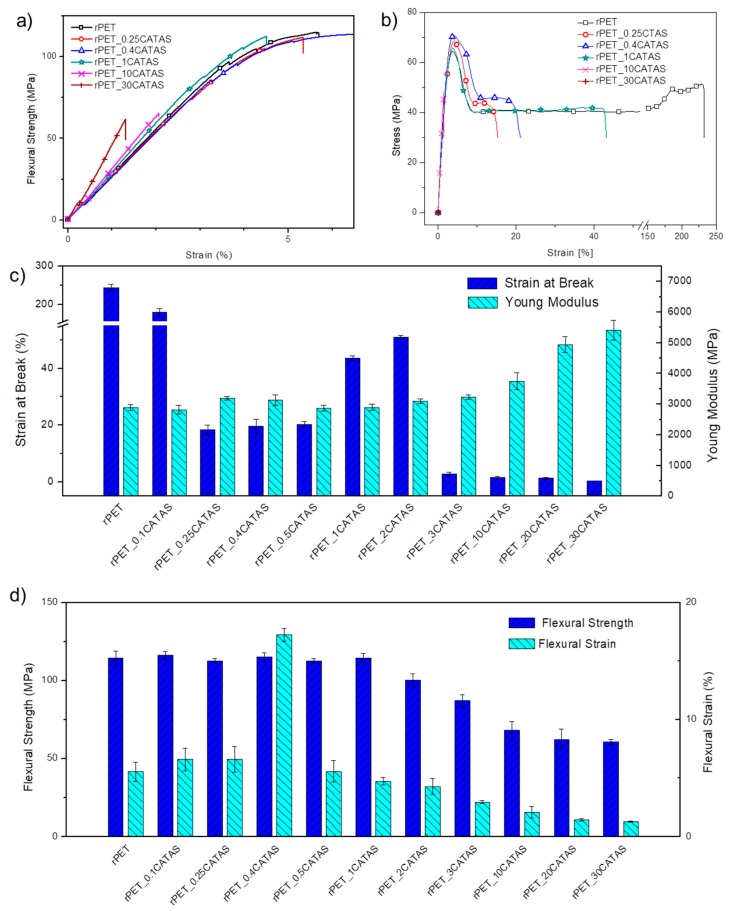
Stress-strain curves from flexural (**a**) and tensile tests (**b**), variation of tensile modulus and strain at break (**c**), variation of flexural stress and strain (**d**) for rPET_CATAS nanocomposites at the different loading levels.

The influence of CATAS presence on tensile and flexural properties of rPET nanocomposites was also investigated. Stress-strain curves for flexural and tensile tests of representative samples are respectively reported in Figure 4a,b, while the variations of the tensile and flexural parameters of all the rPET_CATAS nanocomposites are included in Figure 4c,d. Complementary information on performed tests are also summarized in Table 3. The results of tensile strength of the different formulations showed that when the CATAS content increased from 0.1 to 0.4 wt. %, tangible changes were observed (from 65.5 to 69.4 MPa) and this improvement can be attributed to strong interaction between the salts and the polymer matrix owing to uniform dispersion of nanoparticles, as further confirmed by following FESEM analysis. Besides, amounts of nanoparticles higher than 0.4 wt. % negatively affects the mechanical properties of the nanocomposites, due to agglomeration of nanoparticles [30]. The possible formation of mechanical percolation networks with the RAF of rPET at interfacial regions with confined rPET matrix could justify these results above 0.4 wt. % of CATAS loading. As already observed by Aoyama et al. for grapheme nanoparticles [9], a mechanical percolation threshold can be estimated at these low loading level (between 1 and 2 wt. % in the case of GNP): a rigid amorphous fraction of rPET in nanocomposites is formed at the rPET/CATAS interface, due to the aromatic interactions between the matrix and the filler, both of which contain a π−π conjugation. Molecular chains of rPET in nanocomposites are clearly confined, in compared with neat rPET: below 0.4% wt. of CATAS loading, the restriction of rPET chains is mostly limited to the interfacial region (i.e., X_raf_), therefore, the stiffness enhancement effect is dictated by simple mixing rule (increasing value for E from 2887 up to 3131 MPa, respectively for neat rPET and rPET_0.4CATAS). Above 0.4 wt. %, the restriction of molecular chains of rPET/CATAS is in turn due to the formation of hybrid mechanical percolation networks of CATAS with the X_raf_ of rPET that geometrically restricts the mobility of rPET chains in rPET. This effect was visible also in the registered values for strain at break, where a visible decrease was noted up to the same level of CATAS amount (0.4 wt. %), after that the values showed an increase, due to the absence of X_raf_ fraction between 0.4 and 2 wt. % of CATAS. When the loading was clearly too high (2–30 wt. %), the rPET nanocomposites became extremely fragile.

Similar trend in terms of mechanical performance was found for the nanocomposites in flexural tests. Below the mechanical percolation threshold, estimated between 1% and 2% for these nanostructured systems, the flexural strength remains constant. In particular, a threshold value of 0.4% CATAS for the formation of a mechanical percolation network is conceivable; this model justifies the improvements in the mechanical characteristics of the rPET_0.4CATAS system. The deformation reaches values two times higher than the matrix, maintaining a good flexural strength. However, flexural strength decreases for composites with higher nanofiller loadings (beyond 0.4 wt. %), which could be due to agglomeration of CATAS and reduced interfacial interaction between rPET and nanofiller [31].

This result can be justified again by the formation of aromatic interactions between the matrix and the filler. At this threshold the effect of CATAS is optimal and an exceptional performance is obtained. At values of reinforcement higher than the 2 wt. % CATAS threshold, the excess filler produces block of molecular flows (due to the mechanical effect at the filler/matrix interface and due to the increasing presence of the rigid amorphous phase, which blocks the structure of the composite limiting the deformability of the samples). In high-filling samples an increase in flexural modulus (see Figure 4a) is obtained but, when the deformability limit becomes excessive, the fragile behavior causes premature failure of the samples.

#### 3.2.3. XRD Analysis

**Figure 5:**
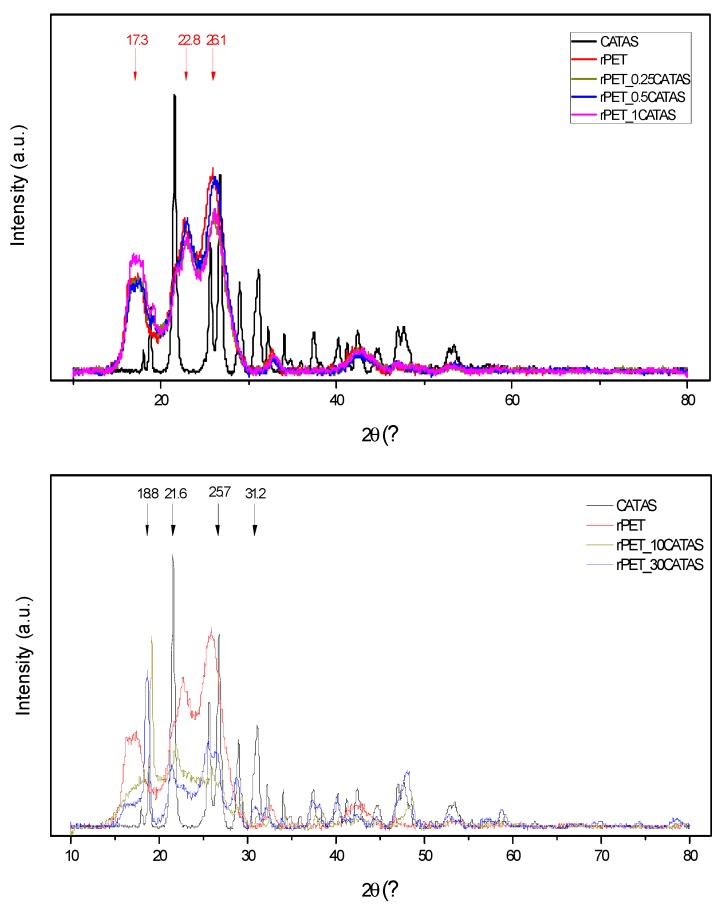
XRD diffraction curves for rPET and rPET/CATAS nanocomposites at low and high loadings.

The XRD patterns of recycled PET and some representative rPET/CATAS nanocomposites (Figure 5) indicate semi-crystalline triclinic structure for rPET matrix. 

Semi-crystalline PET typically shows characteristic crystalline XRD peaks at 2θ = 17.3, 21.7, 22.8, 26.1 and 32.5°, corresponding to the (010), (111), (110), (100), (021), (002) and (101) crystal planes [32]. Although some minor peaks are observed as the amount of CATAS increased in the sample, the intensities of the peaks for rPET decreased in presence of increasing amount of salts, while in parallel the signals related to the CATAS increased, indicating that polymer and salts maintained their original structure [33].

#### 3.2.4. Morphological Analysis

**Figure 6:**
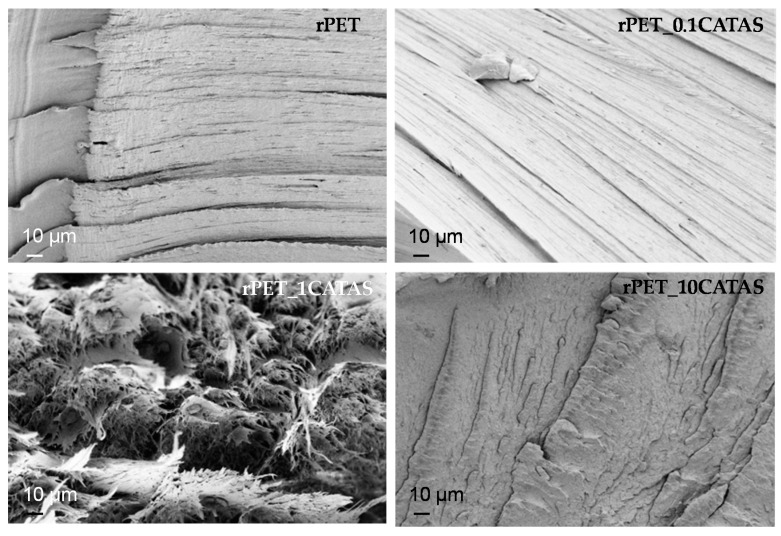
FESEM images of fractured surfaces for rPET and rPET/CATAS nanocomposites.

The morphology of the samples was investigated by using FESEM analysis, performed on the fractured surfaces of the specimens after the tensile test. The SEM images of unreinforced rPET and rPET_CATAS nanocomposite samples at different loading levels are presented in Figure 6. It can be seen a gradual transition from ductile fractured surface of rPET to essentially fragile morphology of rPET_10CATAS, while at intermediate fraction (1 wt. % CATAS) the failure behavior changes, becoming partially brittle. This observation is consistent with the increasing content of amorphous fraction at higher concentrations: this fraction can act to prematurely fracture the composite, reducing the stiffness of the composite, due to CATAS role as stress concentration sites, facilitating crack initiation [34].

#### 3.2.5. Thermal Characterization

**Figure 7:**
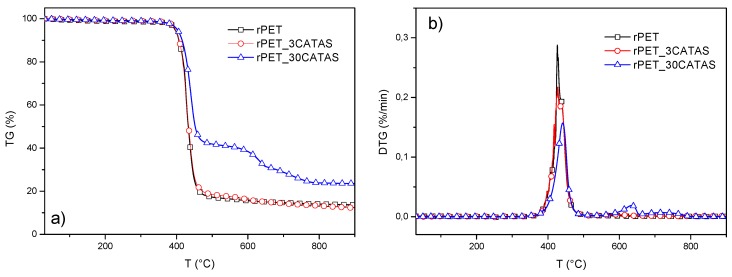
TG curve (**a**) and DTG curve (**b**) for rPET and rPET/CATAS nanocomposites at 3 and 30 wt. % of CATAS.

TGA: The TG weight loss curves and first derivative TG curves for reference rPET and the nanocomposite samples under nitrogen atmosphere are presented in Table 4 and Figure 7a,b. Neat rPET exhibited a single-step decomposition profile: the main degradation process during TGA analysis under nitrogen atmosphere is due to the combined degradation processes—polymer chain degradation through end group initiated mechanism and the thermal degradation of the products formed during polymer chain degradation [35]. The thermal decomposition of polymer is leading to thermally stable cross-linked carbonaceous species which are not undergoing any further decomposition due to the presence of inert nitrogen atmosphere and hence the large amounts of residue at the end of TGA analysis. The addition of different amount of CATAS enhanced the thermal stability of the matrix to some extent (peak temperature shifted from 426 °C for neat rPET to 427 and 441 °C for rPET_3CATAS and rPET_30CATAS), respectively: nevertheless, all the formulations presented a degradation curve similar to the matrix, with a superposition, in the thermal degradation pattern, due to the concomitant decomposition of the salts [36]. The values of residual mass at the end of the tests, measured as 13.7%, 12.3% and 23.6% for neat rPET, rPET_3CATAS and rPET_30CATAS, respectively, are in line with the expected values, according to the registered weight loss for CATAS (43% at the end of the test) already measured in Figure 2b. The decreased residual mass for the rPET_3CATAS system at the end of the test, in comparison with rPET, can be justified by considering that in addition to polymer decomposition, the presence of the alkaline earth metal-based MOF catalyzed the thermal degradation of the neat matrix, lowering the values for final mass residue.

**Figure 8:**
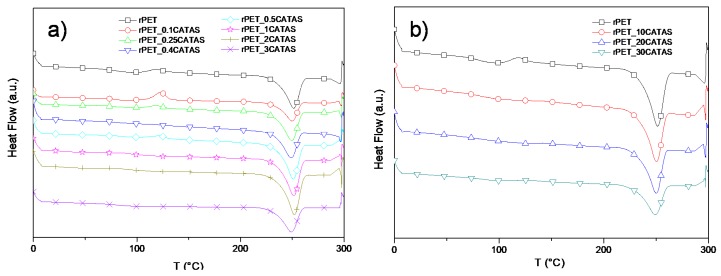
DSC heating scan for rPET and rPET/CATAS nanocomposites at low (**a**) and high (**b**) loading levels.

DSC: DSC heating curves for neat rPET and rPET_CATAS nanocomposites, reported in Figure 8a (low loading) and Figure 8b (high loading), registered the presence of three main events during the heating scan: a stepwise endothermic changes for glass transition at around 76 °C, an exothermic peak for cold crystallization at 117 °C, and one endothermic peak for crystal melting at 250 °C [9]. The peak heat flow for cold crystallization with very low quantity of CATAS nanocomposites become larger compared with that of neat rPET, indicating that CATAS play the role of nucleating agents for crystallization in rPET_0.1CATAS and rPET_0.25CATAS nanocomposite samples. Furthermore, the peak shapes of PET-based nanocomposites became wider, indicating that the nanocomposite cold crystallization process takes a longer time to complete than for neat rPET. This crystallization peak is negligible in rPET_0.4CATAS and becomes again visible in rPET_0.5CATAS as in rPET_0.25CATAS. Despite the variability observed across samples in terms of the cold crystallization behavior, it was observed for all of the samples that the crystallinity level is almost the same for all the nanocomposites with a slight increase at higher filler content.

**Figure 9:**
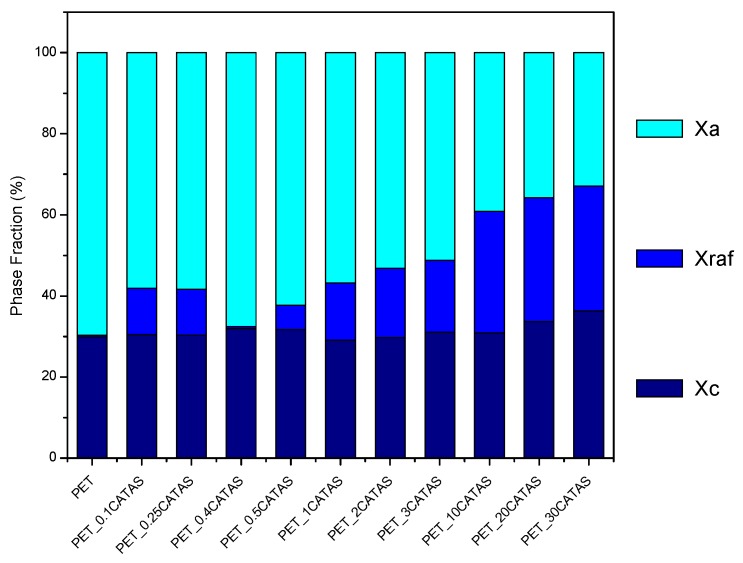
Evolution of different phase fractions for rPET and rPET_CATAS content.

The presence of CATAS, in addition to the nucleating effect, produces a blocking effect of the amorphous phase causing the increase of the fraction of the rigid amorphous phase. At the lower percentages of filler, the nucleating effect is prevalent on the blocking so that the X_raf_ remains lower than 12%. It was hypothesized that the singularity of the rPET_0.4CATAS composite consists of a condition of equilibrium between the nucleating effect of the crystalline phase and the blocking effect of the amorphous phase, coordinating the terephthalate structure of the salts with the terephthalic chains of the matrix. This situation produces a slight increase of X_c_ with a reduction of X_raf_ to values close to rPET neat. The progressive increase of filler in the subsequent formulation bring to a prevalence of the blocking effect and then to a progressive increase of X_raf_. The formation of an important fraction of a rigid amorphous phase limits the formation of crystals as opposed phenomena, hindering the peak of cold crystallization. In addition, during the heating, melting and reconstitution of crystallites reach to an equilibrium between the phases that is influenced by many factors as time, temperature, heating rate, etc. In our systems, a three-phase model show the phase ratio reported in Figure 9 that helps to give a comprehensive explanation of the mechanical results [37,38].

As already observed, flexural test at a speed rate of 2 mm/min shows a large strain for rPET and rPET_0.4CATAS that indeed have a very similar phases ratio with the addition of a possible interfacial slipping effect of the filler in the composite. Tensile test carried out at higher speed (5 mm/min) shows an increase in strength at low amount of CATAS, but it is necessary a larger percentage of filler to obtain the slipping effect at this strain rate. The blocking effect of X_raf_ show its effect even on moduli that increased progressively with the CATAS as soon as the composite become fragile at higher amounts of filler.

## 4. Conclusions

Preparation, by melt extrusion, of CATAS reinforced rPET matrix for nanocomposites loaded at different levels was reported. Results from thermomechanical characterization indicated that CATAS content can be tailored, opening the use of these materials in unexplored industrially relevant applications that require high strength and thermal stability at high temperatures. 

In detail, it was observed that when the CATAS content increased from 0.1 to 0.4 wt. %, an increase for tensile strength and elastic moduli was observed. Nevertheless, a threshold weight amount (0.4 wt. %) of CATAS was also found, by formation at low loading, of a rigid amorphous fraction at the rPET/CATAS interface, that restricted the mobility, as shown by G’ values measured at temperature below and above Tg temperature. When the threshold was surpassed, a restriction of rPET/CATAS molecular chains mobility was also detected, due to the formation, of mechanical percolation networks in addition to physical restrictions (hydrogen and π-π bonding). Additionally, enhanced thermal stability of CATAS filled rPET was registered at a high content due to chemical compatibility between synthetized Ca–Metal Organic Framework and the rPET matrix, rich in stable aromatic rings.

Given the chemical affinity of Ca-based salts and the rPET matrix and the simplicity of the processing methodology necessary to make these systems, this work can pave the way for the facile preparation of similar mixed-matrix materials.

## Figures and Tables

**Table 1:** Developed formulations based on rPET and CATAS.

Sample Name	rPET [%] wt.	CATAS [%] wt.
rPET	100	---
rPET_0.1CATAS	99.9	0.1
rPET_0.25CATAS	99.75	0.25
rPET_0.4CATAS	99.6	0.4
rPET_0.5CATAS	99.5	0.5
rPET_1CATAS	99	1
rPET_2CATAS	98	2
rPET_3CATAS	97	3
rPET_10CATAS	90	10
rPET_20CATAS	80	20
rPET_30CATAS	70	30

**Table 2:** Calculated values of tan δ and G’ at 50 and 150 °C for the rPET/CATAS nanocomposites.

MATERIAL	G’ @ T = 50 °C (×10^9^ Pa)	G’ @ T = 150 °C (×10^8^ Pa)	T_g_ (at tan δ peak) (°C)
rPET	0.89	0.61	90.41
rPET_1CATAS	1.00	0.68	90.48
rPET_10CATAS	1.19	0.96	90.89
rPET_20CATAS	1.47	1.54	91.29
rPET_30CATAS	1.92	2.76	93.43

**Table 3:** Tensile parameters of rPET_CATAS nanocomposites at the different loading levels.

MATERIAL	Young’s Modulus (MPa)		Stress (MPa)		Strain at Maximum Stress (%)		Stress at Break (MPa)		Strain at Break (%)	
rPET	2887	±178	65.5	±3.6	3.99	±0.21	41.0	±5.2	244.2	±16.9
rPET_0.1CATAS	2819	±283	61.7	±4.2	3.68	±0.23	41.7	±3.1	178.9	±20.7
rPET_0.25CATAS	3186	±115	68.9	±2.3	3.98	±0.28	41.9	±1.9	18.3	±3.6
rPET_0.4CATAS	3131	±342	69.4	±1.7	3.89	±0.27	42.4	±3.2	19.7	±4.5
rPET_0.5CATAS	2867	±201	66.8	±0.1	3.90	±0.28	39.6	±0.5	20.2	±2.3
rPET_1CATAS	2898	±206	67.4	±2.5	3.78	±0.26	43.2	±1.7	43.6	±1.6
rPET_2CATAS	3099	±145	64.8	±1.4	3.12	±0.38	39.4	±0.5	51.0	±0.9
rPET_3CATAS	3225	±148	61.9	±8.8	2.91	±0.36	61.8	±8.8	2.9	±1.1
rPET_10CATAS	3751	±549	45.5	±14.1	1.60	±0.42	45.5	±14.1	1.6	±0.7
rPET_20CATAS	4943	±517	36.5	±10.2	1.34	±0.51	36.5	±10.3	1.3	±0.5
rPET_30CATAS	5408	±645	0.3	0.0	0.04	0.01	0.3	0.0	0.3	±0.1

**Table 4:** TGA parameters of rPET_CATAS nanocomposites at three representative loading levels (0, 3 and 30 wt. %).

Material	T_on_ (°C)	T_20%_ (°C)	T_max_ (°C)	Residual Mass (%) (at 900 °C)
rPET	350	417	426	13.7
rPET_3CATAS	350	418	427	12.3
rPET_30CATAS	335	439	441	23.6
